# Molecular epidemiological study of enteroviruses associated with encephalitis in children from Hangzhou, China: Erratum

**DOI:** 10.1097/01.md.0000508480.01540.dc

**Published:** 2016-12-02

In the article “Molecular epidemiological study of enteroviruses associated with encephalitis in children from Hangzhou, China”,^[1]^ which appeared in Volume 95, Issue 40 of *Medicine*, Dr. Wei Li's name was originally misspelled. The article has since been corrected online.

## References

[1] Wei L Qiong Z Xiao-ting S Molecular epidemiological study of enteroviruses associated with encephalitis in children from Hangzhou, China. *Medicine*. 2016 95:e4870. 10.1097/MD.0000000000004870 PMC5059043 27749541

